# Effectiveness of a group educational intervention – prolact - in primary care to promote exclusive breastfeeding: a cluster randomized clinical trial

**DOI:** 10.1186/s12884-022-04394-8

**Published:** 2022-02-16

**Authors:** María Jesús Santamaría- Martín, Susana Martín-Iglesias, Christine Schwarz, Milagros Rico-Blázquez, Julián Alexander Portocarrero-Nuñez, Laura Diez-Izquierdo, Laura Llamosas-Falcón, Ricardo Rodríguez-Barrientos, Isabel Del-Cura-González, Grupo PROLACT, Francisca Martín-Llorente, Leopoldo Casero-Perona, Paz Plasencia-Plasencia, Marta Yolanda Sánchez-Méndez, Pilar Santamaría-Medrano, Rebeca Mielgo-Salvador, Carolina Cañón-Cañón, Dolores Valor-Sánchez, Ramona Fernández-Fernández, Soledad López-Lozano, Marisol Morales-Montalva, María Elena Pérez-Mañanes, Isabel Durand-Rincón, Sara Valdecantos-Coscollano, Verónica Hernández-Hernández, Maria Dolores Noceco-Paredes, Clara Malde-García, Ma Ángeles Miranda-Martín, Nuria de la Peña Antón, Elena Martín-Díaz, Dolores Robas-García, Manuel Parra-Moro, María Concepción Ruiz-del-Castillo, Maria Jesús Geijo-Rincón, Carmen Rivero-Garrido, María Gema Alameda-Hernández, Miriam González-Macías, Isabel Coghen-Vigueras, Raquel Arenas-Yaguez, Carolina San Pablo-Campos, Rosa M Prados-Bueno, Ma Adoración Bejarano-López, Natividad García-Ruiz, Gema Magdaleno-Del-Rey, Lucía Tirado-Jiménez, Ma Jesús Santamaria-Martín, Asunción Reviriego-Gutierrez, Beatriz Soto-Almendro, Paula García-Romero, Elena Zarco-Cid, María Villa-Arranz, Alma Mejía Fernández-de-Velasco, Laura Anta-Rodríguez, María Isabel Sánchez-Prieto-Emmanuel, Luz Divina Barrios-García, Carmen Lozano-Adeva, Luis Mariano Casado-García, Lourdes Gómez-Pérez, Ma Dolores Martínez-Sierra, Martha Olga Escobar-García, Ma Dolores Guerra-Nieto, Elena Azcona-Domínguez, María Ángeles Delgado-Domínguez, Rafael Verdugo-Hernández

**Affiliations:** 1 Centro de Salud Lucero, Gerencia Asistencial de Atención Primaria, Servicio Madrileño de Salud, Madrid, Spain; 2 Dirección Asistencial Sur.Gerencia Asistencial de Atención Primaria, Servicio Madrileño de Salud, Madrid, Spain; 3 Escuela Nacional de Sanidad, Instituto de Salud Carlos III, Madrid, Spain; 4 Research Unit. Gerencia Asistencial Atención Primaria. Servicio Madrileño de Salud, Calle San Martín de Porres 6, 28035 Madrid, Spain; 5 Health Services Research on Chronic Patients Network (REDISSEC), RICORS (RICAPPS) Instituto de Salud Carlos III, Madrid, Spain; 6 Nursing Department, Faculty of Nursing, Physiotherapy and Podiatry, Universidad Complutense de Madrid, Madrid, Spain; 7 Instituto de Investigación Sanitaria Gregorio Marañón (IISGM), Madrid, Spain; 8 Preventive Medicine. Hospital Infanta Sofia. San Sebastian de los Reyes, Madrid, Spain; 9 Preventive Medicine and Public Health, Hospital Universitario, 12 de Octubre, 28041 Madrid, Spain; 10 Department Medical specialties and Public Health, Universidad Rey Juan Carlos, Alcorcón, Madrid, Spain

**Keywords:** Breast-feeding, Primary healthcare, Health education, Clinical trial, Health promotion

## Abstract

**Background:**

The rates of exclusive breastfeeding at 6 months in Spain are far from recommended by the World Health Organization, which is 50% by 2025. Evidence of the effectiveness of group interventions in late postpartum is limited. The objective of this study was to evaluate the effectiveness of the PROLACT group educational intervention for increasing the proportion of mother-child dyads with exclusive breastfeeding at 6 months compared to the usual practice in primary care.

**Method:**

Multicentre cluster randomized clinical trial. A total of 434 mother-child dyads who breastfed exclusively in the first 4 weeks of the children’s life and agreed to participate were included. The main outcome was exclusive breastfeeding at 6 months. Secondary variables were type of breastfeeding, reasons for abandonment, degree of adherence and satisfaction with the intervention. To study the effectiveness, the difference in the proportions of dyads with exclusive breastfeeding at 6 months was calculated, and the relative risk (RR) and number needed to treat (NNT) were calculated with their 95% CIs. To study the factors associated with the maintenance of exclusive breastfeeding at 6 months, a multilevel logistic regression model was fitted. All analyses were performed to intention to treat.

**Results:**

The percentage of dyads with exclusive breastfeeding at 6 months was 22.4% in the intervention group and 8.8% in the control group. PROLACT intervention obtained an RR =2.53 (95% CI: 1.54–4.15) and an NNT = 7 (95%CI: 5–14). The factors associated with exclusive breastfeeding at 6 months were the PROLACT intervention, OR = 3.51 (95%CI: 1.55–7.93); age > 39 years, OR = 2.79 (95%CI: 1.02–7.6); previous breastfeeding experience, OR = 2.61 (95%CI: 1.29–5.29); income between 500 and 833.33 €, OR = 3.52 (95%CI 1.47–8.47).); planning to start work before the infant was 6 months old, OR = 0.35 (0.19–0.63) .

**Conclusions:**

The PROLACT intervention in primary care is more effective than the usual practice for maintaining exclusive breastfeeding at 6 months, and can therefore be considered evidence-based practice for implementation in standard practice.

**Trial registration:**

The trial was registered with ClinicalTrials.gov under code number NCT01869920 (03/06/2013).

**Supplementary Information:**

The online version contains supplementary material available at 10.1186/s12884-022-04394-8.

## Introduction

### Breastfeeding and its benefits

Breastfeeding is the natural way of feeding children and provides the food that is best adapted to the nutritional needs of the child [1]. The World Health Organization (WHO) recommends exclusive breastfeeding as a public health strategy during the first 6 months of life, followed by the introduction of complementary feeding with continued breastfeeding until the child is 2 years old [1]. It proposes the global goal that by 2025, at least 50% of mothers will exclusively breastfeed their children for the first 6 months [2]. Three types of breastfeeding are defined: i) exclusive breastfeeding, which includes the feeding expressed breast milk or milk from a wet nurse to which oral rehydration solution, drops, syrups, vitamins, minerals or medicines are added; ii) predominant breastfeeding, which includes breastfeediing plus water or water-based drinks and/or fruit juices; and iii) complementary feeding, which includes any solid or liquid food, including milk of non-human origin and child formula, in addition to breast milk [3].

Breastfeeding is a natural act and a learned behaviour that most mothers can perform; however, it is favoured by the presence of accurate information and support within the family, community and health care system [1].

Breastfeeding provides numerous maternal and child health benefits. A systematic review that included 28 meta-analyses and systematic reviews showed that breastfeeding is associated with a decreased risk of sudden death, necrotizing enterocolitis and other childhood diseases as well as a significant reduction in child mortality in low-income countries. The study also supports the association of breastfeeding with a decreased risk of breast cancer in the mother [4]. Several studies in Organisation for Economic Co-operation and Development countries also show that breastfeeding is associated with significant savings for national health systems via a reduction in the incidence of childhood diseases and a possible decrease in maternal diseases [5–7], estimating these savings for the Spanish National Health System to be more than 5.6 million euros for each point of increase in the breastfeeding rate during 2014 [8].

### Prevalence of breastfeeding and associated factors

Although the initiation of breastfeeding is a mainstream occurrence in almost all countries, there is a progressive decline throughout the first months of life. Data published in 2016 by UNICEF [9] indicate that worldwide, only 43% of children receive exclusive breastfeeding at 6 months. These highest rates (43%) are found in countries in South Asia and eastern and southern Africa. However, the European region has the lowest rate of all WHO regions at 25% [10]. In Spain, the 2006 National Health Survey shows that there was 68.4% exclusive breastfeeding at 6 weeks and 24.72% at 6 months. In 2011, there was a slight increase to 28.53%, and in 2017, there was a further increase to 39% [11].

In the Spanish context, there are multiple factors that promote breastfeeding, with older age and a university education of the mother, greater weight of the child at birth and delivery at term standing out; in contrast, tobacco consumption by the mother is associated with a lower probability of breastfeeding [12]. Regarding why mothers do not initiate breastfeeding, 34% indicated an immediate return to work, and 32% reported a lack of support from health professionals; for early abandonment of breastfeeding, the most frequent reasons were the sensation of low milk production (29%) and returning to work (18%) [13]. Another barrier is the commercial promotion of breast milk substitutes in the context of widespread public and professional acceptance of equivalence of breast milk substitutes and breastfeeding [14–16]. Given these obstacles, it is essential that health professionals develop the skills and attitudes needed to teach and support women to breastfeeding and maintain exclusive breastfeeding during the first 6 months of their children’s lives.

### Strategies and interventions to improve breastfeeding rates

The WHO and UNICEF have introduced the Baby Friendly Initiative, which aims to encourage health centres and health services to adopt practices that protect, promote and support exclusive breastfeeding from birth. The main guidelines for preventive activities and health promotion include recommendations to promote exclusive breastfeeding in primary care (PC). Evidence in support of these guidelines comes mostly from individual interventions [17]. The Cochrane review and meta-analysis of McFadden et al. 2019 [18] included 63 studies that evaluated educational interventions to promote breastfeeding; 3 of these studies evaluated group interventions without associated individual interventions. One of them was conducted in Sydney with Vietnamese women [19] (a population group in Australia with especially low breastfeeding rates); the results indicated increases in levels of breastfeeding knowledge, attitudes and intention. In the second intervention, which included women in Brazil who were pregnant with twins [20], the intervention did not significantly affect the rates of breastfeeding, and in the third, a group intervention in mothers in Croatia [21] that consisted of self-directed training, an increase in breastfeeding was observed at the time of the intervention. More studies are needed on the effectiveness of group breastfeeding interventions in different contexts.

The Madrid Health Service, operating within the Portfolio of Standardized Services offered by Primary Care, has implemented individual and group interventions to promote breastfeeding [22]. Educational interventions in this context are carried out at the health centre level; therefore, to study the effect of clustering, cluster designs are needed. The group interventions include an educational intervention designed by a multidisciplinary team of breastfeeding experts that uses breastfeeding promotion as a means to improve maternal and child health. This intervention is directed towards the 263 health centres of the Madrid Region, which serve a population of 6,498,560.

The main objective of the study was to evaluate the effectiveness of the PROLACT group educational intervention compared with the usual practice in PC health centres for increasing the proportion of mother-child dyads engaging in exclusive breastfeeding at 6 months. The secondary objectives were to evaluate the effectiveness of the intervention for maintaining any type of breastfeeding at 6 months and to describe women’s adherence and degree of satisfaction with the group educational intervention.

## Materials and methods

### Design

A community-based, multicentre, parallel clinical trial was designed with randomization of 6 months of follow-up clusters. The methodology is described in detail in the study protocol [23] . In the preparation of the publication, the CONSORT CLUSTER guidelines were followed [24].

### Setting and study population

The study was carried out in mother-child pairs at 10 health centres in the Community of Madrid (Spain), which provide health coverage to a population of 2,043,460. Mother-child dyads of women ≥18 years old and their children born at term (≥ 37 weeks of gestation) with a birth weight ≥ 2.5 kg who attended the health centres for any reason during the first 4 weeks of the children’s life and who breastfed exclusively between 01/26/2015 and 06/30/2016 were included consecutively. The mothers had to be able to communicate in Spanish to follow the requirements of the study. Participating mothers provided written informed consent. We excluded dyads with mothers who were participating in other clinical trials, could not attend follow-up visits, had clinical contraindications to breastfeeding (tuberculosis; chickenpox; herpes lesions in the breast; Chagas disease; HIV; human T-lymphotropic virus (HTLV) I and II; drug dependence; treatment with radioactive isotopes, chemotherapeutic agents or antimetabolites) and/or whose children had clinical conditions that hinder, prevent or contraindicate breastfeeding (orofacial malformations, galactose-1-phosphate uridyltransferase deficiency).

### Sample size

To calculate the sample size, we believe that the educational group intervention can increase the proportion of mother-child dyads that use exclusive breastfeeding at six months by 15%. Assuming that 24% of mothers use exclusive breastfeeding at six months (National Health Survey (2006). National Statistics Institute http://www.ine.es), a type I error of 5% and a strength of 80%, we need a sample of 150 mother/infant pairs in each group. As this is a randomized design with conglomerates (each conglomerate is a health centre), the sample size needs to be increased taking into account the effect of the design (ED = 1+ (ñ-1)*ICC, where ñ is the average size of each cluster and ICC is the intra-class coefficient of correlation). Considering an ICC of 0.01 and an average size of 30 pairs per centre [25], the effect of the design is 1.29. Taking this data into account, the sample size increases to 194 pairs in each group. If we then take into account the loss rate of 10%, the total size surpasses 432 pairs (216 for each branch) (doi: 10.1186/s12884-018-1679-3).

### Randomization and blinding

An independent statistician conducted random assignment to form groups of the same size from the list of participating health centres using Epidat 3.1. Available at: Epidat 3.1 (Español) - Consellería de Sanidade - Servizo Galego de Saúde (sergas.es). Subsequently, mother-child dyads were selected within each unit by consecutive sampling until the number needed for the cluster was reached. Due to the nature of the intervention, neither the mothers nor the health professionals could be blinded. The analysis was performed by researchers who did not know the participants’ allocation.

### Variables

The main outcome variable was exclusive breastfeeding at 6 months reported by the mother, based on the WHO guidelines for how the child had been fed in the 24 h prior to the interview. As secondary outcome variables, the type of feeding at 6 months (exclusive breastfeeding, predominant breastfeeding and complementary feeding) [3] and the duration of exclusive breastfeeding (days) were collected. Variables related to the mother and child were also recorded. For the mother, sociodemographic variables (age, education level, income level, nationality, work situation, cohabitation with the partner), obstetric history (pregnancies, abortions, live births, type of delivery, previous breastfeeding experience) and lifestyle variables (weight, height, and tobacco consumption) were collected. For the child, sex, birth weight, discharge from the hospital with the mother, APGAR score, separation from the mother during the hospital stay and whether the child was breastfed during the first hours after delivery were collected.

The reasons for breastfeeding abandonment were collected with a questionnaire specially designed for the study by the research team. Adherence to the intervention was measured as the number of group education sessions attended by the dyad (adequate adherence was considered attendance of at least 85% of the planned sessions [22]). Degree satisfaction survey of the Workshop about educational intervention group to promote BF breastfeeding on the Madrid Health Service, based on the SERVQUAL model, commonly used in our setting. It has scores ranging from 19 to 190, with 19 indicating minimum satisfaction and 190 indicating maximum satisfaction [22].

### Data collection

The nurse or paediatrician collected the baseline variables (1 month postpartum) of the mother and child during consultation after the informed consent form was signed. Five follow-up visits were made at 2, 3, 4, 5 and 6 months after delivery in the control group and intervention group. The visits at 2, 4 and 6 months coincided with the standards of the healthy child protocol and the childhood vaccination schedule of the Community of Madrid, and the visits at 3 and 5 months were made by telephone to inquire about the infant’s feeding during the previous 24 h. The information was recorded in an electronic data collection logbook and confidentiality, anonymity, and compliance with current regulations were ensured.

### Intervention

Intervention group: The complex PROLACT intervention is an educational group intervention based on a breastfeeding workshop designed by the expert group of the General Directorate of Primary Healthcare of the Madrid Health Department. Its objectives are the acquisition, reinforcement and/or consolidation of the knowledge and skills needed to initiate and maintain exclusive breastfeeding and the development of a positive attitude regarding breastfeeding. A total of six weekly group sessions of 120 min each were conducted. It consists of theoretical and practical content, active participation of the mothers in discussion groups and the learning of skills through the direct practice of breastfeeding.

This intervention was developed in accordance with the recommendations and taxonomy proposed by the Cochrane Effective Practice and Organisation of Care Review Group. The intervention is described in detail in Additional File 1 following the approach proposed by Perera et al. [26] and the template for intervention description and replication (TIDieR) (see Additional File 2).

Control group: This group received advice regarding the promotion of breastfeeding and the benefits of exclusive breastfeeding in individual consultations according to clinical practices described in the portfolio of standardized services of the Community of Madrid [22].

### Statistical analysis

The database was filtered before the statistical analysis was performed to improve the quality of the data collected. The use of a cluster design was taken into account in all phases of the analysis.

Descriptive analysis (means, medians, frequencies of distribution) of the demographic and baseline characteristics of the dyads in both groups was performed. In addition, we compared the baseline characteristics of the dyads in the 2 groups. Student’s t test or the Mann-Whitney test was used if the normality hypothesis was rejected for the data. If the study variables were qualitative, Pearson’s chi-squared test or Fisher’s exact test or Z test difference proportions were used when applicable.

The results for the primary outcomes were subjected to an intention to treat (ITT) analysis. Missing values for the main outcome variables were added using the last observation carried forward (LOCF) method. Between-group differences in the proportions of exclusive breastfeeding at 6 months in the mother and child dyads were calculated using Fisher’s exact test, and confidence intervals were estimated. The relative risk (RR) and the number needed to treat (NNT) with 95% CI were calculated for exclusive breastfeeding.

To study the factors associated with maintaining exclusive breastfeeding at 6 months, a multilevel logistical regression model was constructed. The dependent variable was exclusive breastfeeding at 6 months, and the independent variable was the treatment group. The model was adjusted for possible confounding factors. Effectiveness was determined using an ITT analysis.

Other secondary analyses included the type of breastfeeding at 6 months, described as percentages with 95% CI, and the reasons for abandonment. In the intervention group, adherence and satisfaction with the intervention were described and measured with a Likert-type scale, and the results are reported along with the corresponding 95% CI. All *p*-values below 0.05 were considered statistically significant for all cases. The STATA 14 software programme was used.

## Results

**Fig. 1:**
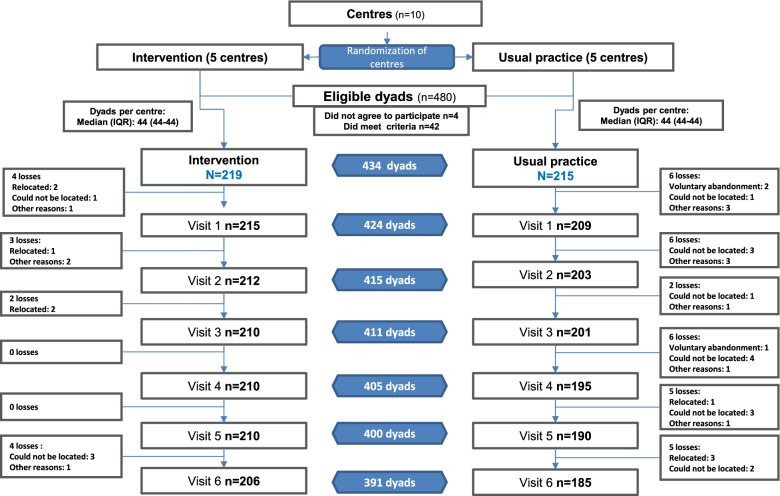
The flow of participants and the reasons for the losses

Between January 2015 and June 2016, a total of 480 dyads were invited to participate in the study; of these, 4 refused to participate, and 42 did not meet the selection criteria. The final sample included in the study comprised 434 dyads (219 in the intervention group and 215 in the control group). A total of 391 (90.1%) dyads completed the 6-month follow-up. The distribution of losses was not proportional between the groups and was higher in the control group (14%) than in the intervention group (5.9%). There were 3 voluntary dropouts in the control group, no one in the intervention group. Most of the losses (69.8%), similar in both groups, were due to failure to locate or relocate the dyad. The flow of participants and the reasons for the losses are presented in Fig. 1.

### Participant characteristics

The mean age of the women who participated in the study was 32.8 (SD ±5) years. A total of 80.2% were of Spanish nationality, 55.1% had a university education, and 72.6% had been actively working prior to delivery. Tables 1 and 2 show the characteristics of the participants.

**Table 1:** Sociodemographic and clinical baseline characteristics of the mother-child dyads

	Total (***n*** = 434)	Control (***n*** = 215)	Intervention (***n*** = 219)	***P***-value
**Sociodemographic characteristics**				
Mother’s age (years)^a^	32. 8 ± 5.0	32.5 ± 5.2	33.2 ± 4.8	0.02
University studies	239 (55.1%)	103 (47.9%)	136 (62.1%)	0.003
Paid work	315 (72.6%)	150 (69.8%)	165 (75.3%)	0.19
Planned return to work before 6 months	246 (78.1%)	115 (76.7%)	131 (79.4%)	0.34
Number of family-unit members^a^	2.5 ± 0.8	2.7 ± 0.8	2.4 ± 0.7	0.002
Living with the partner	411 (94.7%)	200 (93%)	211 (96.4%)	0.12
Has other children	184 (42.4%)	106 (49.3%)	78 (35.6%)	0.004
Dysfunctional family APGAR score	11 (2.5%)	5 (2.3%)	6 (2.7%)	0.78
Income/household member^b^	833.3 [500–1250]	750 [500–1167]	833.3 [750–1250]	< 0.001
Spanish	348 (80.2%)	166 (77.2%)	182 (83.1%)	0.12
**Clinical characteristics**				
Maternal BMI (kg/m2)^a^	24.8 ± 4.1	25.3 ± 4.4	24.4 ± 3.7	0.05
Maternal smoking	31 (7.1%)	13 (6.0%)	18 (8.2%)	0.38
Gestational age (weeks)^a^	39 ± 1.2	39 ± 1.1	39 ± 1.2	0.54
Diseases/clinical problems in pregnancy^c^	94 (21.7%)	50 (23.2%)	44 (20.1%)	0.42
Caesarean delivery	88 (20.3%)	41 (19.1%)	47 (21.5%)	0.54
Sex of child (male)	212 (48.8%)	111 (51.6%)	101 (46.1%)	0.25
Apgar score at 1 minute^a^	8.9 ± 1	8.8 ± 1	9.0 ± 1	0.56
Weight at birth (kg)^a^	3.3 ± 0.4	3.3 ± 0.4	3.3 ± 0.4	0.30
Days of admission of the child^a^	3 ± 1.3	3 ± 1.5	3 ± 1	0.30

^a^mean ± SD ^b^median [IQR]. Family members not including the child of the study. ^c^risk of abortion, risk of premature birth, heart disease, diabetes, hypertension, others

*BMI* body mass index

**Table 2:** Promotion, perception and experience of breastfeeding

	Total (n = 434)	Control group (n = 215)	Intervention group (n = 219)	***P***-value
***Promotion***				
Use of any type of pacifier	169 (38.9%)	99 (46.0%)	70 (32.0%)	0.003
Breastfeeding in the first 2 h postpartum	346 (79.9%)	163 (75.8%)	183 (83.9%)	0.04
Use of nipple shields	79 (18.2%)	33 (15.3%)	46 (21%)	0.13
Support during pregnancy	318 (73.4%)	148 (68.8%)	170 (78%)	0.03
Family breastfeeding support	110 (25.4%)	62 (28.8%)	48 (22%)	0.10
Mother was breastfed	346 (79.7%)	35 (16.3%)	53 (24.2%)	0.04
Professional breastfeeding support	93 (21.5%)	42 (19.5%)	51 (23.4%)	0.33
Preparation for delivery by a midwife	254 (58.7%)	119 (55.3%)	135 (61.9%)	0.16
Made the decision to breastfeed				0.11
*Before pregnancy*	335 (77.4%)	171 (79.5%)	164 (75.2%)	
*During pregnancy*	83 (19.2%)	34 (15.8%)	49 (22.5%)	
*Postpartum*	15 (3.5%)	10 (4.7%)	5 (2.3%)	
Person who most influenced the decision				0.001
No one *	215 (49.7%)	123 (57.2%)	92 (42.2%)	
Partner *	129 (29.8%)	49 (22.8%)	80 (36.7%)	
*Another family member*	44 (10.2%)	26(12.1%)	18 (8.3%)	
*Health personnel*	28 (6.5%)	12 (5.6%)	16 (7.3%)	
*Others (including Breastfeeding support groups)*	17 (3.9%)	5 (2.3%)	12 (5.5%)	
***Perception***				
Mother’s belief that Breastfeeding is better than formula feeding	424 (97.9%)	209 (97.2%)	215 (98.6%)	0.34
***Previous experience***				
Breastfed another child	178 (41%)	102 (47.4%)	76 (34.7%)	0.01
Breastfed for at least 6 months	91 (21%)	53 (24.7%)	38 (17.4%)	0.06

(*) categories whose difference was significant

A total of 94.7% of the mothers lived with a partner, and the median family income was approximately 833 € (IQR 500–1250) per member of the family unit.

A total of 20.3% of the women had a caesarean delivery, and 87.6% underwent delivery and follow-up at health centres in the public network of the National Health System.

A total of 184 women (42.4%) were multiparous; of these, 178 (96.7%) had breastfed a previous child, and 51.1% had breastfed for a minimum of 6 months.

Regarding attitudes toward breastfeeding, 424 women (97.9%) stated that breastfeeding was better than formula feeding, more than 70% had received some type of promotion or information related to breastfeeding during pregnancy, and 79.9% breastfed within the first 2 h after delivery. There were no multiple births or separation of dyads at the time of birth.

Some baseline differences were found between the 2 groups: the mothers in the intervention group were 0.7 years older than those in the control group; the percentage of primiparous mothers was 64.4% in the intervention group compared to 50.7% in the control group; and only one-third of the children in the intervention group used pacifier, compared to almost half of the children in the control group. A higher percentage of women in the intervention group breastfed in the first 2 h after delivery (83.9% versus 75.8%) and had been exposed to breastfeeding promotion during pregnancy.

### Primary outcome

At the 6-month follow-up, 77.2% (95% CI: 71.6–82.7) of the mothers in the intervention group maintained some type of breastfeeding, compared to 58.2% (95% CI: 51.5–64.7) of the mothers in the control group. Regarding the main result, 22.4% (95% CI: 16.9–27.9) of the mothers in the intervention group maintained exclusive breastfeeding, compared to 8.8% (95% CI: 5.04–12.63) in the control group. The RR was 2.53 (95% CI: 1.54–4.15) at 6 months. The NNT of the PROLACT intervention was 7 (95% CI: 5–15).

Table 3 shows the exclusive breastfeeding results during the first 6 months; without adjusting for any factor, significant differences were observed in favour of the intervention group at all months; the maximum difference occurred in the 4th month, with 24.35% more dyads in the intervention group engaging in exclusive breastfeeding.

**Table 3:** Exclusive breastfeeding during the 6 months follow-up: differences between the intervention and control groups

Visit/ month	Exclusive Breastfeeding (%) Intervention Group	Exclusive Breastfeeding (%) Control Group	Difference Exclusive Breastfeeding (%) 95% CI	RR 95% CI	NNT 95% CI
First	92.7	80	12.7 (6.33–19.06)	1.16	8 (5–16)
				(1.07–1.25)	
Second	82.2	63.7	18.5 (10.29–26.65)	1.29 (1.15–1.45)	5 (4–10)
Third	74	56.3	17.7 (8.88–26.51)	1.31 (1.14–1.51)	6 (4–11)
Fourth	66.2	41.9	24.4 (15.25–33.45)	1.58 (1.32–1.90)	4 (3–7)
Fifth	44.8	27	17.78 (8.9–2.67)	1.66 (1.27–2.16)	6 (4–11)
Sixth	22.4	8.8	13.5 (6.84–20.23)	2.53 (1.54–4.15)	7 (5–15)

When the results were adjusted for age, educational level and economic level, the PROLACT intervention was associated with a higher proportion of mothers who engaged in exclusive breastfeeding, OR = 1.57 (95%CI: 1.02–2.41).

In the empty model, the variability explained by the cluster was 0.14; this decreased to 0.049 when the intervention and adjustment variables were introduced in the model.

The median odds ratio (MOR) among centres was 1.48. This can be interpreted as the MOR of exclusive breastfeeding of the mother-child dyads at different health centres (comparing the higher-risk centres to the lower-risk centres). The MOR of 1.48 was lower than the intervention OR, suggesting that variation among health centres contributed less to exclusive breastfeeding at 6 months than the intervention did. Table 4 shows the factors associated with maintaining exclusive breastfeeding at 6 months.

**Table 4:** Factors associated with exclusive breastfeeding at 6 months

	Odds ratio	p	95%CI
PROLACT intervention	3.51	0.003	1.55–7.93
Mother’s age			
*18–29 years*	1.83	0.095	0.9–3.74
*30–39 years (ref)*	1		
*> 39 years*	2.79	0.045	1.02–7.6
Income per family member			
*< 500 euros/person (ref)*	1		
*500–833.33 euros/person*	3.52	0.005	1.47–8.47
*> 833 euros/person*	2.15	0.051	0.996–4.65
Previous breastfeeding	2.61	0.01	1.29–5.29
Use of any type of pacifier	0.58	0.099	0.3–1.11
Plan to return to work before 6 months	0.35	0.001	0.19–0.63

The factors that were associated with maintaining exclusive breastfeeding at 6 months were having received the PROLACT intervention, 3.5 (95%CI: 1.5–7.93); being older than 39 years, OR = 2.78 (95%CI: 1.02–7.6),; and previous breastfeeding experience, OR = 2.6 (95%CI: 1.29–5, 29). The probability of exclusive breastfeeding at 6 months was decreased among those who planned to return to work before the child was 6 months old, OR = 0.35 (95% CI 0.19–0.63) and among those with an income less than 500 euros per member of the family unit (excluding the child of the study).

### Secondary outcomes

**Fig. 2:**
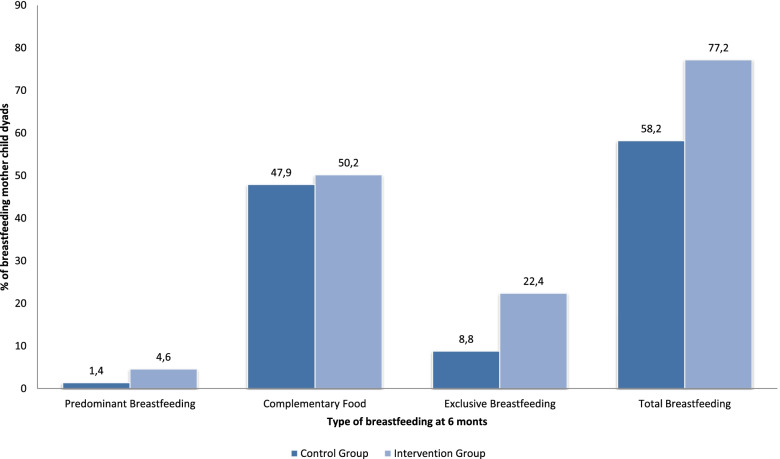
Prevalence of the different types of breastfeeding after 6 months in the intervention and control group

At the 6-month follow-up, 4.6% (95% CI 2.5–8.3%) of the mothers in the intervention group maintained predominant breastfeeding, compared to 1.4% (95%CI: 0.4–4.3) in the control group; and the 50.2% (95%CI: 43.6–56.8%) of the mothers in the intervention group maintained complementary feeding, compared to 47.9% (95%CI: 41.3–54.6%) in the control group (Fig. 2). The most frequent reasons for abandonment that the women reported were their own decision to stop breastfeeding (56.2%) and returning to work (33.7%).

Among the mothers in the intervention group, 64.5% attended at least 85% of the scheduled sessions, the limit defined as good adherence, and the median satisfaction indicated on the satisfaction scale (min 0 - max 190) was 176 points (IQR: 160–186).

## Discussion

### Main findings of the studies

The educational group intervention implemented at the health centres increased the percentage of dyads with exclusive breastfeeding at 6 months by 3.5 times (95%CI: 1.55–7.93) compared to usual practice. Maternal age between 30 and 39 years, lower income per member of the family unit and planning to return to work before 6 months were associated with a lower probability of maintaining exclusive breastfeeding.

### Comparisons with other studies

The exclusive breastfeeding rates at 6 months obtained in our study (22.4%) are lower than those based on data from national health surveys (24.7%) [11] and those reported in other local and national studies [27], a difference that can be explained by the disparity of measurement methods and definitions used in our context. In this study, the WHO’s recommendations for the measurement of exclusive breastfeeding rates were strictly followed. Each month, the mothers were asked either by phone or in person at the PC consultation if, in the previous 24 h, their children had been given water or water-based drinks; if they had, the dyads was automatically registered as engaging in predominant breastfeeding, even if it was an isolated situation, and this may explain the low rate of exclusive breastfeeding in our study. On the other hand, in Spain, data collection with epidemiological surveys that incorporate breastfeeding is not performed periodically and does not always use the indicators and methodology recommended by the WHO [3]. Instead, these data are obtained by asking the mothers of children under 5 years of age at the time of the survey what type of breastfeeding they had received based on the WHO definitions described above. This method may be condition a possible recall bias [28]. In our region, according to data from the health report of the Community of Madrid in 2012, which uses electronic medical records of PC as a source [29], the rate of exclusive breastfeeding and predominant breastfeeding at 6 months is 17.9%. This report did not use the WHO definitions, nor did it identify exclusive breastfeeding independently.

Regarding breastfeeding in any forms, it should be noted that control group’s rate of 9% is similar to the rates reported in the Community of Madrid Health Report 2012 [29].

The results regarding the efficacy of the intervention for the maintenance of exclusive breastfeeding and for any some type of breastfeeding at 6 months are higher than those obtained in the studies included in the systematic review by McFadden et al. [18]. A group educational breastfeeding intervention for mothers of twins increased the probability of breastfeeding by 87% during the first 30–40 days but produced a breastfeeding rate of only 39.4% compared to 37.9% in the control group at 6 months [20]. The rate of exclusive breastfeeding after six months was of only 7,7% in the counseling group and 9,1% in the control group. In a study of Vietnamese women in Australia [19], group intervention resulted in a difference of 24.4% in the percentage of breastfeeding at 4 months, but this difference was reduced to 9.5% at 6 months. It is important to note that both studies reported prenatal interventions and. A postnatal group intervention such as the one used in the present study can more easily address doubts and problems that may arise during breastfeeding, which may explain the differences in the effects of the interventions at 6 months. In all cases, additional face-to-face support leads to higher rates of exclusive breastfeeding at 6 months.

Evaluating the effect of the intervention on total breastfeeding, we observed an RR of 1.33 (95%CI: 1.16–1.52), with 77.2% breastfeeding in the intervention group compared to 58.2% in the control group at 6 months, percentages similar to those obtained in other observational studies conducted in different regions of our country (Fig. 2).

The results reflect, first, the influence of the mother’s return to work on maintaining exclusive breastfeeding. Maintaining exclusive breastfeeding EBF can have negative consequences for job stability and professional advancement especially in women between 30 and 39 years of age. In addition, the legislation could have played a role in the decision about breastfeeding. At the time of the study, women in Spain were basically entitled to a period of suspension from work and a maternity benefit of sixteen weeks. In addition, workers had the right to a one-hour leave from work that they could divide into two fractions when it was intended for feeding their child under the age of nine months [30].

The greater proportions of exclusive breastfeeding among women older than 39 years who give birth may correspond to personal maternity options that imply a more favourable context for exclusive breastfeeding.

In our study, education level and smoking habits, factors that are traditionally associated with breastfeeding, were not associated when the results were adjusted for the intervention and economic level. It is likely that while interventions to promote breastfeeding, such as the one implemented in the present study, can support changes in habits and facilitate the training of mothers, they cannot modify income level, a factor that also clearly conditions mothers’ return to work.

Previous exclusive breastfeeding experience is expected to predispose patients to future exclusive breastfeeding, and the use of pacifier has been identified in different studies as a factor that decreases the probability of breastfeeding. However, as experts indicate, we should focus on providing objective information about the time and effort required for breastfeeding and help women to set challenging and achievable goals, according to their current situation [31].

### Strengths and limitations

One strength of this study is its methodological rigor. It followed the methodology and definitions of the types of breastfeeding recommended by the WHO and was conducted in the context of a pragmatic cluster RCT that allowed us to study the effectiveness of the intervention in real conditions of practice [32].

Among the limitations and biases of our study, it should be noted that although the centres were randomly assigned, there were baseline differences between groups, with the women in the intervention group having a higher income and education level and less use of pacifier. This difference may be due to the differences observed among centres. In the empty model, the centre explained 14% of the variability in exclusive breastfeeding; this was reduced to 4.9% after adjustment for the effect of the intervention and the remaining adjustment variables. It could also be related to the fact that a better socioeconomic situation favoured acceptance of the intervention (e.g., by making it possible to attend group sessions), although the percentages of acceptance were similar.

On the other hand, recruitment was performed prior to randomization, and the dyad was required to start follow-up upon inclusion. This may have led the professionals to offer invitations to participate that were conditioned by the mother’s profile, which could also have contributed to a selection bias. The motivation of the professional who monitored the dyad could not be controlled in any of the groups, a factor that may have interfered with the effect of the intervention. The participation of both centres and professionals was voluntary; thus, those that were more motivated to promote breastfeeding may have been more motivated to participate in the study. However, to homogenize and control for this effect, no centre that had a BFI accreditation was incorporated, and if this bias existed, it would have affected both the intervention group and the control group.

As this is a nonpharmacological intervention, there may be differences in the way in which different professionals conduct it. To minimize these differences, the professionals who provided the intervention participated in a 20-h basic BF training course proposed by the baby Friendly initiative and received training in group education techniques [27].

All these limitations may have conditioned a better result in the intervention group and led to overestimation of the effect; however, given the magnitude of the effect found, it would not have conditioned the relevance of the result.

### Implications of the study findings

As stated in the Clinical Practice Guideline on Breastfeeding in the Spanish National Guideline Plan (GUIASALUD), “the recommendations of all interventions that obtain an RR > 2 can be adapted as a health policy in most situations” [33, 34]; the PROLACT intervention has obtained this effect.

## Conclusion

The PROLACT intervention in primary care is more effective than the usual practice for maintaining exclusive breastfeeding and any type of breastfeeding at 6 months. Women’s adherence and degree of satisfaction with the group educational intervention were high.

PROLACT intervention can therefore be considered evidence-based practice for implementation in standard practice.

## Supplementary Information

**Additional File 1.** Description of intervention.

**Additional File 2.** Template for Intervention Description and Replication (TIDieR).

## Data Availability

The datasets generated and/or analysed during the current study are not publicly available due Regarding data exchange, the Ethics Committee approved this research without considering the option of data sharing. However, they are available from the corresponding author on reasonable request. Each new project based on these data must be previously submitted to Ethics Committee for approval.

## Abbreviations

RR: Relative Risk
NNT: Number Needed to Treat
WHO: World Health Organization
UNICEF: United Nations Children’s Fund
HIV: Human Immunodeficiency Virus
HTLV: Human T-lymphotropic Virus
ED: Effect of the design
ICC: Intraclass Correlation Coefficient
ITT: Intention to Treat
LOCF: Last observation carried forward
CI: Confidence Interval
SD: Standard Deviation
N: Number in the sample
IQR: Interquartile Range
BMI: Body Mass Index
OR: Odds Ratio
MOR: Median Odds Ratio
