# Supplementary material for: Effectiveness of a group educational intervention – prolact - in primary care to promote exclusive breastfeeding: a cluster randomized clinical trial
Source: BMC Pregnancy Childbirth. 2022 Feb 16;22:132. doi: 10.1186/s12884-022-04394-8 (PMC8851786; doi:10.1186/s12884-022-04394-8)
Supplement: Supplementary file 1 — Additional File 1. Description of intervention. [file 12884_2022_4394_MOESM1_ESM.docx]

Additional File 1. Description of intervention

| **Time line** | **Intervention** | | | **Control** |
| --- | --- | --- | --- | --- |
| Professional recruitment | \| 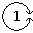 \|  \|  \|  \| \| --- \| --- \| --- \| --- \| | | | \| \| 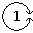 \|  \|  \|  \| \| --- \| --- \| --- \| --- \| \|  \| \| --- \| --- \| --- \| --- \| --- \| --- \| |
| Training session for professionals | 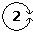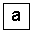 | | 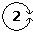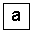 | |
| Randomization | Healthcare centers randomised allocation | | | |
| Training on PROLACT intervention | 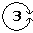 |  | | |
| Dyads recruitment and baseline visit | \|  \|  \|  \| 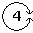 \|  \| \| --- \| --- \| --- \| --- \| --- \| | | | \|  \| 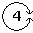 \| \| --- \| --- \| |
| Intervention: mother-child dyads | \| 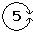 \| 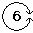 \| \| --- \| --- \| | | | \|  \| 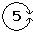 \|  \| \| --- \| --- \| --- \| |
| Follow-up and outcome measurements | monthly measurement of objectives up to month 6 | | | |

| 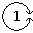 | Recruitment of professionals to participate in the study. |
| --- | --- |
| 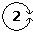 | Training session for professionals: 3 hours. Presentation for the project. Training in good practices in research and electronic data capture systems. |
| 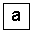 | Delivery of written documentation about the project |
| 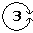 | Breastfeeding training for professionals of the intervention group.  20 hours of basic breastfeeding training. |
| 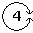 | Recruitment of dyads. Informed consent. Collection of baseline variables in both groups. |
| 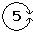 | Usual care: Portfolio of Standardized Primary Care Services |
| 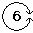 | PROLACT intervention.  6 weekly sessions of 2 hour of duration. |
