# Supplementary material for: Effectiveness of a group educational intervention – prolact - in primary care to promote exclusive breastfeeding: a cluster randomized clinical trial
Source: BMC Pregnancy Childbirth. 2022 Feb 16;22:132. doi: 10.1186/s12884-022-04394-8 (PMC8851786; doi:10.1186/s12884-022-04394-8)
Supplement: Supplementary file 2 — Additional File 2. Template for Intervention Description and Replication (TIDieR). [file 12884_2022_4394_MOESM2_ESM.docx]

Additional File 2. Template for Intervention Description and Replication
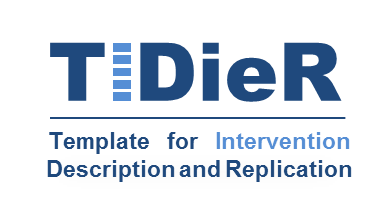
 (TIDieR)

| **Item number** | **Item** | **Where located** | |
| --- | --- | --- | --- |
|  | PROLACT: Breastfeeding education group intervention | Primary  paper | Other |
|  | **Abbreviated name** |  |  |
| **1.** | PROLACT: Breastfeeding education group intervention | page 8 |  |
|  | **Why** |  |  |
| 2. | Rates of exclusive breastfeeding up to 6 months in Spain are far from the target recommended by the World Health Organization. The main objective of the PROLACT study was to evaluate the effectiveness of a group breastfeeding education intervention at primary care health centres for increasing the proportion of mother-child dyads with exclusive breastfeeding at 6 months in a cluster-randomized trial. Among the secondary objectives were to evaluate the effectiveness of the intervention for maintaining any type of breastfeeding at 6 months and to describe the adherence and degree of satisfaction of women who participated in the educational group intervention. | page 8 |  |
|  | **What** |  |  |
| **3 and 4.** | **Intervention group:** This was a didactic group intervention based on breastfeeding workshops designed by an expert group from the General Directorate of Primary Health Care of Madrid. Its objectives were the acquisition, reinforcement and/or consolidation of the knowledge and skills necessary to initiate and maintain exclusive breastfeeding for child feeding and the development of a positive attitude towards breastfeeding. The activities began around the first month of the child’s life (the point at which breastfeeding abandonment is greatest, according to existing studies) and continues for 6 weeks. Mothers were offered the opportunity to attend the training sessions with the person who had the greatest influence on their decision to breastfeed (social support).  **Control group:** The usual practice includes providing individual advice on the benefits of exclusive breastfeeding during the child’s first 6 months of life and the introduction of complementary foods afterwards. Mothers had to visit the health centre at least twice: once before the infant was 6 months old and another when he or she was between 6 and 12 months old. | pages 11,12 | Supplement 1 |
|  | **Who** |  |  |
| **5.** | Health professionals who were trained in breastfeeding provided the intervention group with 20 hours of basic training in breastfeeding according to the Baby Friendly Initiative (BFI). |  | Supplement 1 |
|  | **How** |  |  |
| **6.** | The intervention consisted of theoretical and practical content. The mothers participated actively in discussions and learned skills through the direct practice of breastfeeding. Follow-up was carried out through periodic reviews, according to the protocol, and through telephone calls to collect the study variables. | pages 11,12 | Supplement 1 |
|  | **Where** |  |  |
| **7.** | Ten health centres in the Community of Madrid (Spain). | page 9 | Supplement 1 |
|  | **When and how much** |  |  |
| **8.** | Six weekly sessions of 120 minutes each. |  | Supplement 1 |
|  | **Adaptations** |  |  |
| **9.** | There is no evidence that the intervention was adapted. | N/A |  |
|  | **Modifications** |  |  |
| **10.^ǂ^** | There are no records of modifications to the intervention. | N/A |  |
|  | **How well** |  |  |
| **11.** | **Planned:** Data regarding adherence to the intervention, measured as the number of group education sessions attended by the dyad, were collected (adequate adherence was considered attendance of at least 85% of the planned sessions). Degree of satisfaction was measured through the short form SERVQUAL survey, which has a range of 19 to 190 in which 19 is minimum satisfaction and 190 is maximum satisfaction. | Page 13 |  |
| **12.^ǂ^** | **Observed:** A total of 61.1% of the mothers had attended at least 85% of the planned sessions, which was defined as good adherence; the median score on the satisfaction scale was 175 points (IQR: 154-186). | Page 18 |  |
